# Consumption of diets with low advanced glycation end products improves cardiometabolic parameters: meta-analysis of randomised controlled trials

**DOI:** 10.1038/s41598-017-02268-0

**Published:** 2017-05-23

**Authors:** Estifanos Baye, Velislava Kiriakova, Jaime Uribarri, Lisa J Moran, Barbora de Courten

**Affiliations:** 1 Monash Centre for Health Research and Implementation, School of Public Health and Preventive Medicine, Monash University, 43-51 Kanooka Grove, Clayton, VIC 3168 Australia; 2 Department of Medicine, The Mount Sinai School of Medicine, One Gustave Levy Place, New York, NY 10029 USA

## Abstract

Studies examining the effects of consumption of diets low in advanced glycation end products (AGEs) on cardiometabolic parameters are conflicting. Hence, we performed a meta-analysis to determine the effect of low AGE diets in reducing cardiometabolic risk factors. Seventeen randomised controlled trials comprising 560 participants were included. Meta-analyses using random effects models were used to analyse the data. Low AGE diets decreased insulin resistance (mean difference [MD] −1.3, 95% CI −2.3, −0.2), total cholesterol (MD −8.5 mg/dl, 95% CI −9.5, −7.4) and low-density lipoprotein (MD −2.4 mg/dl, 95% CI −3.4, −1.3). There were no changes in weight, fasting glucose, 2-h glucose and insulin, haemoglobin A1c, high-density lipoprotein or blood pressure. In a subgroup of patients with type 2 diabetes, a decrease in fasting insulin (MD −7 µU/ml, 95% CI −11.5, −2.5) was observed. Tumour necrosis factor α, vascular cell adhesion molecule-1, 8-isoprostane, leptin, circulating AGEs and receptor for AGEs were reduced after consumption of low AGE diets with increased adiponectin and sirtuin-1. Our findings suggest that diets low in AGEs may be an effective strategy ﻿for﻿ improving cardiometabolic profiles in individuals with and without type 2 diabetes.

## Introduction

Advanced glycation end products (AGEs) are formed endogenously from the non-enzymatic reaction of sugars with proteins or lipids^1^. These processes are present in healthy individuals but are accelerated in diabetes due to high glucose levels with patients with diabetes having approximately 50% higher levels of serum AGEs than those of healthy, age-matched controls^2–4^. In diabetes, AGEs contribute to both micro- and macrovascular complications^5, 6^. Consumption of highly heated processed foods typical of a Western diet also increases the level of circulating AGEs^2^. Globalisation and industrialised methods of food processing have dramatically altered diets, increasing exposure to AGEs, which are used in foods to impart desirable properties such as longer shelf life, sterility, flavour and colour^7, 8^. The average total daily adult consumption of AGEs in our diet is around 16,000 AGE kU/day and if the diet is rich in heat-processed food, grilled or roasted meat, and high in sugar content, this could lead to an AGE intake exceeding 20,000 kU/day^1, 9, 10^. Of the AGEs ingested, 10–30% are absorbed in the intestine. Approximately one third of all AGEs consumed are excreted by the kidneys or via faeces, leaving two thirds to be retained by the body, adding to the total AGE load^11^. Patients with renal disease, regardless of diabetic status, have significantly elevated circulating levels of AGEs in part due to the impaired renal clearance of AGEs^5, 12^. CKD induces a state of high oxidative stress and inflammation that will increase the endogenous production of AGEs^12^.

Dietary AGE intake can be readily reduced by lower cooking temperatures, shorter cooking times, higher humidity and moisture content, and by the use of acidic (low pH) ingredients^1, 2^. A high AGE diet has been shown to enhance chronic inflammation, oxidative stress, endothelial dysfunction, and thereby increase the risk of development of T2DM and its complications^13, 14^. Consumption of low AGE diets has been suggested to be a promising intervention in preventing chronic diseases^10, 13–15^. Several clinical trials showed that restriction of high AGE intake reduced cardiometabolic risk factors and disease^10, 15–17^. However, there are currently discrepancies in the literature regarding the effect of a low or high AGE diet based on participants’ health status (T2DM, CKD or healthy) or by amount of reduction of AGEs in the diet. Prior systematic reviews assessing the effect of low AGE and high AGE diets on insulin resistance, markers of inflammation and oxidative stress^13, 14^ have not considered other measures of glucose homeostasis and cardiovascular risk factors. More importantly, the previous reviews did not conduct meta-analysis for clinical cardiometabolic parameters. The aim of this study was therefore to conduct a meta-analysis on the effects of dietary AGEs (dAGEs) on a broad range of cardiometabolic parameters in individuals with or without T2DM.

## Results

### Search Results

**Figure 1:**
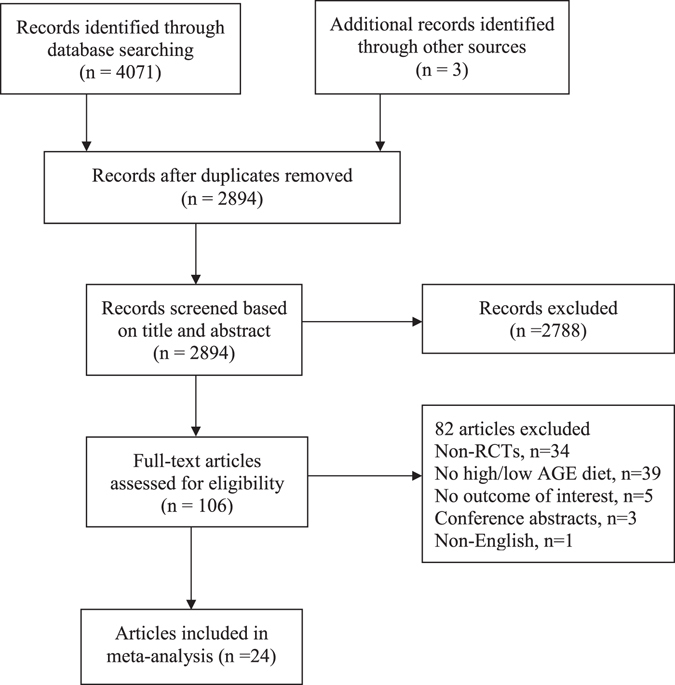
Flow chart of the process of study selection: schematic diagram shows the study selection process.

A total of 4074 records were retrieved from the searches. After excluding duplicates (n = 1180), the remaining 2894 articles were screened for eligibility based on title and abstract. From these, 106 articles were screened for eligibility from full-text. Of these, only 24 articles from 17 studies that met the inclusion criteria were selected (Figure 1).

### Characteristics of included studies

The detailed characteristics of all included studies are described in Supplementary Table 1. All of the authors were contacted for additional information; half of them provided the requested data^16–23^. Ten parallel^17, 19–29^ and seven randomised crossover studies^10, 16, 18, 22, 30–37^ were included. The studies were conducted at research centres^10, 15, 16, 18, 24, 27, 34–37^, hospitals^20, 28, 30, 32, 33^ and universities^17, 19, 21–23, 25, 26, 29, 31^ in USA^17, 20–24, 27–29^, Australia^16, 18^, Denmark^19, 31^, Germany^10, 34–37^, Mexico^25, 26^, Spain^30, 32, 33^ and France^15^. Twelve studies involved people without T2DM^15, 17, 18, 21, 23, 26–33^, and six were in patients with T2DM^10, 17, 22, 24, 25, 34–37^. The majority (n = 13) of the studies used high AGE diets which were two or more times higher in AGE content than the low AGE diets^1, 10, 15, 17–19, 22–24, 27–37^ and the rest used high AGE diets that were less than two times higher in AGE content than the low AGE diets. The duration of 11 studies was ≥4 weeks^15, 17, 19–29^, four for 2–4 weeks^16, 18, 22, 30, 32, 33^ and two for <2 weeks^10, 31, 34–37^.

### Risk of bias assessment

The risk of bias assessment is presented in Supplementary Table 2. Seven studies reported an adequate randomisation process using computer generated random numbers^16, 18, 19, 25, 26, 31^ while the rest did not report on their randomisation process. The majority (n = 10) did not describe methods of allocation concealment^10, 15, 17, 20–25, 27–30, 32–37^. Only two studies indicated that participants’ were blinded throughout the study period (participants were masked to the allocation of the diet type and to how the diet might affect glucose metabolism)^16, 27^ whereas the remaining studies were not blinded^10, 15, 17–26, 28–30, 32–37^. In addition, seven studies described that investigators and care providers as well as outcome assessors were blinded^10, 16, 18, 25–27, 32, 34–37^. Intention-to-treat analyses were used in eight studies^10, 17, 18, 21–24, 29, 31, 34–37^. All of the crossover studies used a washout period of two or more weeks for interventions that lasted for 2 weeks or more^16, 18, 22, 30–33^ except one study which used a 10 days washout period after a 4 week intervention^15^. Overall, five studies were rated with low risk of bias^16, 18, 26, 27, 31^, three with moderate risk of bias^10, 19, 23, 25, 34–37^ and eight with insufficient information^15, 17, 20–24, 28–30, 32, 33^.

## Effects of dietary AGEs on cardiometabolic parameters

### Anthropometric and blood pressure measurements

There was no significant difference between low and high AGE diets with regards to weight (MD −0.8 kg, 95% CI −4.4 to 2.9)^16, 18, 19, 21–23, 26–29^, body mass index (BMI) (MD −0.5 kg/m^2^, 95% CI −1.9 to 0.9)^18, 19, 23, 24, 26–29^, waist circumference (MD −1.2 cm, 95% CI −3.7 to 1.53)^19, 23, 26^, systolic (MD 1.6 mmHg, 95% CI −2.1 to 5.2) and diastolic (MD 1.8 mmHg, 95% CI −0.8 to 4.4) blood pressure^22, 23, 26, 29^ (Table 1). Subgroup analyses also did not reveal any differences in these measurements between the two diets with regards to participant’s T2DM status, amount of dietary AGEs and length of follow-up (Supplemental Table 3).

**Table 1:** Meta-analysis of low AGE and high AGE diets for cardiometabolic parameters.

Parameters	Overall effect				Studies (n)	Sample size
	MD	95% CI	I^2^	P		
Weight (kg)	−0.8	−4.4, 2.9	31	0.68	10	424
BMI (kg/m^2^)	−0.5	−1.9, 0.9	38	0.49	8	355
Waist Circumference (cm)	−1.2	−3.7, 1.3	0	0.34	3	202
Systolic BP (mm Hg)	1.6	−2.1, 5.2	0	0.40	4	213
Diastolic BP (mm Hg)	1.8	−0.8, 4.4	0	0.17	4	213
Fasting glucose (mg/dl)	−0.4	−2.4, 1.7	27	0.73	10	550
2-h Glucose (mg/dl)	−7.2	−16.7, 2.3	0	0.13	2	113
HbA1C (%)	−0.01	−0.09, 0.08	0	0.97	4	266
Fasting Insulin (µU/ml)	−2.4	−4.9, 0.14	73	0.06	5	367
2-h Insulin (µU/ml)	0.3	−1.5, 2.1	0	0.76	3	113
Insulin AUC	−20.3	−57.1, 16.5	0	0.28	2	139
HOMA-IR	−1.3	−2.3, −0.2	68	0.02	5	261
Total Cholesterol (mg/dl)	−8.5	−9.5, −7.4	0	<0.0001	5	254
LDL (mg/dl)	−2.4	−3.4, −1.3	0	<0.0001	6	279
HDL (mg/dl)	−1.6	−6.6, 3.3	71	0.52	7	379
Triglycerides (mg/dl)	−7.7	−20.1, 4.8	49	0.23	8	419
TNFα (ng/mg)	−4.7	−7.0, −2.3	73	0.0001	6	234
CRP (mg/dl)	−0.06	−0.4, 0.2	47	0.67	4	75
Adiponectin (µg/ml)	7.0	5.6, 8.4	65	<0.0001	3	154
Leptin (ng/ml)	−18.6	−29.1, −8.2	69	0.0005	2	90
VCAM-1 (ng/ml)	−314.5	−506.9 −122.1	60	0.001	5	174
8-Isoprostane (pg/ml)	−110.3	−168.9, −51.6	19	0.0002	4	157
eGFR (ml/min/173 m^2^)	1.5	0.7, 2.2	48	0.0002	2	139
Serum CML (U/ml)	−6.2	−8.7, −3.7	74	<0.0001	7	228
Serum MG (nmol/ml)	−0.6	−0.9, −0.3	71	0.0001	7	228
sRAGE (mRNA)	−241.0	−356.4, −125.8	56	<0.0001	4	157
AGER1 (mRNA)	41.2	−29.7, 112.0	75	0.26	4	157
SERT1 (mRNA)	188.5	133.6, 243.3	0	<0.0001	3	108
P66 (mRNA)	−31.0	−51.4, −10.8	28	0.003	2	67

Random effects model was used. AGER1, advanced glycation endproduct receptor 1; BMI, body mass index; BP, blood pressure; CI, confidence interval; CML, carboxymethyl lysine; CRP, c-reactive protein; eGFR, estimated glomerular filtration rate; HDL, high density lipoprotein; HbA1c, haemoglobin A1c; HOMA-IR, homeostatic model of insulin resistance; Insulin AUC, insulin area under the curve; MD, mean difference; MG, methylglyoxal; P, p-value; SERT1, sertoli cell protein 1; sRAGE, soluble form of receptor for advanced glycation end products.

### Measures of glucose homeostasis

#### Glycaemic control

There was no significant difference between low and high AGE diets with regards to fasting glucose (MD −0.4 mg/dl, 95% CI −2.4 to 1.7)^15, 17–19, 22–24, 26, 27, 29^, 2-h glucose (MD −7.2 mg/dl, 95% CI −16.7 to 1.9)^10, 19^, and HbA1c (MD −0.01%, 95% CI −0.09 to 0.08)^15, 17, 23–25^ (Table 1). It was not possible to conduct meta-analysis for glucose area under the curve (AUC) as there was only one study that reported on this measure^23^.

Subgroup analyses did not reveal any differences in fasting glucose irrespective of participants’ T2DM status, amount of dietary AGEs and length of follow-up. No evidence of difference was shown in HbA1c based on participants’ T2DM status. However, subgroup analysis for HbA1c for dietary AGE content and length of follow up, and 2-h glucose for all subgroups was not possible as there was no enough studies (less than 2) in each groups (Supplemental Table 3).

#### Insulin resistance and secretion

Insulin resistance (measured by homeostatic model of insulin resistance (HOMA-IR)) was significantly reduced in the low AGE compared to the high AGE diet (MD −1.3, 95% CI −2.32 to −0.24)^16, 17, 19, 21, 23^ (Table 1). However, there was no significant difference in fasting insulin (MD −2.4 µU/ml, 95% CI −4.9 to 0.4)^15, 17, 19, 23–25^, 2-h insulin (MD 0.2 µU/ml, 95% CI −1.54 to 1.9)^10, 19^ and insulin AUC after oral glucose tolerance test (OGTT) (MD −20.3 µU/ml, 95% CI −57. 1 to 16.5)^16, 23^ between the diets (Table 1). Meta-analysis was not performed for M-value measured by glucose clamp during the intravenous glucose tolerance test as they were only reported by one study^16^, and for other insulin sensitivity measures such as Matsuda, QUICKI, fasting C-peptide and HOMA-B which weren’t reported by any studies.

The findings from the subgroup analyses showed that low AGE diets reduced HOMA-IR regardless of participants’ T2DM status (non-T2DM: MD −0.5, 95% CI −1 to −0.03; T2DM: MD −4.9, 95% CI −5.5 to −4.3) and length of follow up, but only in participants who consumed low AGE diets of two or more times less in AGE contents compared to high AGE diets. Low AGE diets decreased fasting insulin only in patients with T2DM (MD −7 µU/ml, 95% CI −11.5 to −2.5) but not in non-T2DM (MD −1.8 µU/ml, 95% CI −4.4 to 0.9). No difference was shown in terms of dAGE content or the length of follow up. Subgroup analysis was not conducted for 2-h insulin and insulin AUC as there was no enough studies reported on this parameter (Supplemental Table 3).

### Cardiovascular risk factors

#### Lipid profile

**Figure 2:**
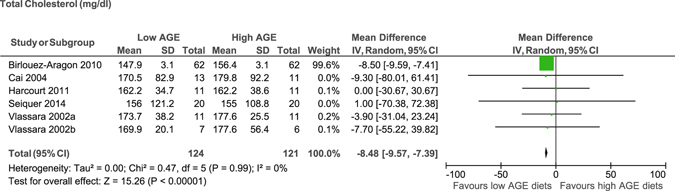
Meta-analysis of low AGE and high AGE diets for total cholesterol (mg/dl): forest plot shows the effect of low AGE and high AGE diets on total cholesterol.

**Figure 3:**
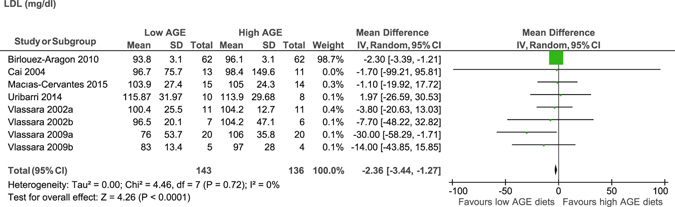
Meta-analysis of low AGE and high AGE diets for low density lipoprotein (mg/dl): forest plot shows the effect of low AGE and high AGE diets on low density lipoprotein.

In the whole group, there was a significant reduction in total cholesterol (MD −8.5 mg/dl, 95% CI −9.5 to −7.4)^15, 18, 22, 24, 25, 32^ (Figure 2) and LDL (MD −2.4 mg/dl, 95% CI −3.4 to −1.3) (Figure 3)^15, 21, 22, 24, 26, 29^ after consumption of low AGE compared to high AGE diets. No evidence of significant difference was observed for HDL (MD −1.6 mg/dl, 95% CI −6.6 to 3.3)^15, 21–24, 26, 29^, and triglycerides (MD −7.7 mg/dl, 95% CI −20 to 4.8)^15, 17, 22–24, 26, 29, 32^ between the diets (Table 1). We could not perform meta-analysis for free fatty acids as there was no study reported on this parameter.

Subgroup analyses revealed total cholesterol was only reduced in patients without T2DM (MD −8.5 mg/dl, 95% CI −9.6 to −7.4) but not in patients with T2DM (MD −5.3 mg/dl, 95% CI −27.6 to 17.1). No statistical difference in LDL was observed based on participants’ T2DM status, however, effect size was similar in both T2DM (MD −3.3, 95% CI −8.3 to 1.8) and non-T2DM (MD −4.3, 95% CI −19 to 11) participants. Both total cholesterol and LDL was also decreased only in those who followed for longer time (4 weeks and above). Participants’ who consumed low AGE diet (2 or more times less in AGE content than high AGE diet) was associated with decreased LDL levels than its counterpart. However, it was not possible to perform subgroup analysis for total cholesterol with respect to dietary AGE content. Furthermore, both HDL and triglycerides did not show difference in terms of participants’ T2DM status, dAGE content and length of follow up (Supplemental Table 3).

### Hard clinical outcomes

None of the included studies reported hard clinical outcomes.

### Inflammatory markers and adipocytokines

**Figure 4:**
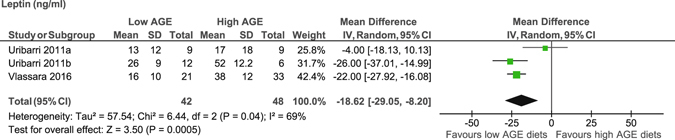
Meta-analysis of low AGE and high AGE diets for leptin (ng/ml): forest plot shows the effect of low AGE and high AGE diets on leptin.

**Figure 5:**
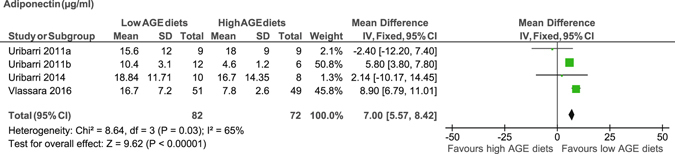
Meta-analysis of low AGE and high AGE diets for adiponectin (µg/ml): forest plot shows the effect of low AGE and high AGE diets on adiponectin.

We showed a significant decrease in tumour necrosis factor (TNFα) in peripheral mononuclear cells (PMNC) (MD −4.7 pg/mg, 95% CI −7 to −2.3)^17, 20–23, 29^, and leptin (MD −18.6 ng/ml, 95% CI −29.1 to −8.2)^17, 23^ (Figure 4) and an increase in adiponectin levels (MD 7 µg/ml, 95% CI 5.6 to 8.4) (Figure 5) after consumption of the low AGE diets compared to the high AGE diets^17, 21, 23^. However, we showed no difference in serum C-reactive protein (CRP) levels (MD −0.06 mg/dl, 95% CI −0.36 to 0.23)^18, 21, 22^ between the diets (Table 1). Only one study reported monocyte chemoattractant protein-1^18^, migration inhibitory factor^18^, interleukin-6^18^, nuclear factor kappa β^18^ and plasminogen activator inhibitor-1 levels^20^, and therefore meta-analysis was not performed.

The findings from the subgroup analyses showed that CRP levels were significantly reduced in T2DM patients (MD −1.22 mg/dl, 95% CI −2.27 to −0.18), but not in non-T2DM (MD −0.01 mg/dl, 95% CI −0.12 to 0.11). CRP levels were not different in response to dAGE content and length of follow up. In addition, low AGE diets were associated with a significant reduction in TNFα regardless of the participants’ T2DM status and dAGE content. Adiponectin levels were also decreased regardless of participants’ T2DM status but only reduced in those who consumed low AGE diet of 2 or more times less AGE content. However, it was not possible to conduct subgroup analysis for TNFα and adiponectin with regards to length of follow-up as all of the studies were followed for 4 or more weeks (Supplemental Table 3).

### Markers of endothelial dysfunction and oxidative stress

Compared to high AGE diets, low AGE diets showed reduction of vascular cell adhesion molecule 1 (VCAM-1) (MD −217.8 ng/ml, 95% CI −313 to −121)^20–23, 29^ and 8-isoprostane (MD −110.3 pg/ml, 95% CI −168.9 to −51.6)^20, 21, 23, 29^ (Table 1). Only one study reported urine isoprostane^31^, ubiquinol^15^, and no studies reported on intracellular adhesion molecule-1 or pulse wave velocity, and thus meta-analysis was not performed.

On subgroup analyses, low AGE diets were associated with decreased VCAM-1 and 8-isoprotane irrespective of the amount of dAGE content. In addition, VCAM-1 was only reduced in patients without T2DM and followed for any duration. However, subgroup analysis was not performed for 8-isoprotanes as all of the included studies were followed for 4 weeks and more (Supplemental Table 3).

### Renal function

Estimated glomerular filtration rate (eGFR) slightly increased after a consumption of low AGE compared to high AGE diets (MD 1.45 ml/min/173 m^2^, 95% CI 0.69 to 2.22)^16, 23^ (Table 1). Due to the limited number of studies (n = 2), subgroup analysis was not possible. We could not conduct meta-analysis for creatinine clearance, proteinuria and urine albumin^18^ as they were reported only by one study.

### Circulating AGEs and transcription factors

**Figure 6:**
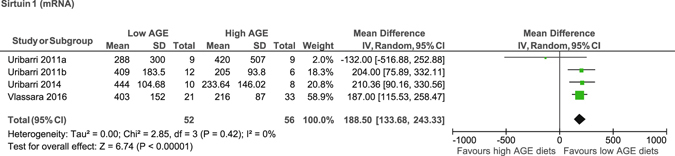
Meta-analysis of low AGE and high AGE diets for sirtuin-1 (mRNA): forest plot shows the effect of low AGE and high AGE diets on sirtuin-1.

The low AGE diet reduced levels of circulating carboxymethyl-lysine (MD −6.2 U/ml, 95% CI −8.7 to −3.7)^17, 21, 23, 24, 26, 28, 29^, methylglyoxal (MD −0.6 nmol/ml, 95% CI −0.9 to −0.3)^17, 21, 23, 24, 26, 28, 29^, and receptor for advanced glycation end products in PMNC (MD −241 mRNA, 95% CI −356, −124)^17, 21, 23, 29^ and p66^shc^ (MD −31 mRNA, 95% CI −51 to −11)^21, 29^ and increased PMNC sirtuin-1 (MD 188.5 mRNA, 95% CI 133, 243)^17, 21, 23^ (Fig. 6) compared to the high AGE diet. No evidence of significant difference was reported for advanced glycation end products receptor −1 (AGER1) in PMNC (MD 41.2 mRNA, 95% CI −29.7, 112)^17, 21, 23, 29^ between the diets (Table 1). Meta-analyses were not possible for urinary and faecal AGE levels as urine carboxyehtyl-lysine^16^, faecal carboxymethyl-lysine^15^, total methylglyoxal^37^ and endogenous secretory receptor for advanced glycation end products^18^ were reported by only one study. Although three studies reported on urine carboxymethyl-lysine^15, 16, 28, 31^ and methylglyoxal levels^16, 28, 31^, but it was impossible to perform meta-analysis due to the differences in measuring and reporting these parameters.

The findings of the subgroup analyses showed that low AGE diets reduced carboxymethyl-lysine, methylglyoxal, receptor for advanced glycation end products and sirtuin-1 regardless of participants’ T2DM status and dAGE content. However, low AGE diets were associated with an increased AGER1 only in T2DM patients (MD 96 mRNA, 95% CI 46 to 146) but not in non-T2DM (MD −20 mRNA, 95% CI −12 to 72) with no difference in AGER1 by dietary AGE content (Supplemental Table 3). It was not possible to perform subgroup analysis with respect to length of follow up as in all studies participants were followed for 4 weeks and above.

## Discussion

This meta-analysis assessed the impact of low AGE versus high AGE diets on cardiometabolic parameters. We have demonstrated that consumption of low AGE diets decreased insulin resistance regardless of participants’ T2DM status and reduced fasting insulin levels in patients with T2DM as well as decreased total cholesterol in participants without T2DM. In addition, low AGE diets compared to high AGE diets reduced TNFα, VCAM-1, 8-isoprostane, leptin, circulating AGE levels, receptor for AGE, and P66^shc^, and increased adiponectin levels and sirtuin-1 in PMNC in both individuals with and without T2DM. A small increase in eGFR was also observed after intake of low AGE diets.

Clarke and colleagues^13^ reported that insulin resistance was improved by low AGE diets with no change in fasting glucose and HbA1c in healthy individuals and patients with T2DM based on the Cohen effect size of few individual studies (not pooled effects). We have extended these findings by pooling the findings of six studies (n = 261 participants) and showed that consumption of low AGE diets significantly improved insulin resistance regardless of participants’ T2DM status and length of follow up. In addition, we have also shown that fasting insulin was reduced in patients with T2DM. This finding may suggest that low AGE diets can decrease fasting insulin only in individuals with higher insulin levels at baseline. Our meta-analyses also showed that fasting glucose, HbA1c, 2-h glucose and anthropometric parameters did not change after consumption of low AGE diets. There is still uncertainty regarding the optimal amount of AGE restriction required to achieve beneficial effects in terms of improvement of insulin resistance reduction. Compared to high AGE diets, the studies which reduced the amount of AGE content in the low AGE diets by at least 50% showed statistically significant results in the subgroup analysis, supporting the theory that the greater the reduction in low AGEs in a standard Western diet, the more pronounced the benefit on insulin resistance. Given that the diets differed in each study, with variable baseline standard diet/AGE levels it is difficult to comment on the optimal absolute amount of AGE reduction needed in everyday diet to achieve significant beneficial results on insulin resistance.

In this study, we have shown that consumption of low AGE diets reduced LDL levels regardless of length of follow up and amount of dAGE content. A similar effect size in LDL levels was also observed in both individuals with and without T2DM although the statistical test was not significant. This could be due to the small number of studies included in each of ﻿the ﻿subgroup analyses. We also showed that total cholesterol was reduced in patients without T2DM after intake of low AGE diets for any duration. These findings were consistent with previous reports^13, 14^ that suggested long-term restriction of AGEs in diet decreases LDL-cholesterol in patients with T2DM and CKD. Although we did not show a change in HDL, triglycerides or blood pressure, these findings still support that low AGE diets may be important for the prevention of cardiovascular diseases through reducing LDL-cholesterol levels.

Our meta-analysis also showed that low AGE diets increased eGFR in individuals without T2DM. This small (mean 1.45 ml/min) clinically non-significant increase resulted from only two studies. The first study in obese individuals showed a significant increase (mean 1.3 ml/min) but lasted only for 2 weeks^16^. The second study did not show any significant changes in eGFR in individuals with metabolic syndrome over a period of 1 year^38^. These results therefore should be interpreted with caution. Further research is warranted to investigate the effects of AGE diet restriction on measures of renal function, including proteinuria, end stage clinical outcomes and progression to renal failure given the high mortality and morbidity of this diabetic complication.

We have also demonstrated that consumption of low AGE diets reduced TNFα, 8-isoprotane and leptin, increased serum adiponectin and PMNC sirtuin-1 regardless of participants’ T2DM status and amount of dietary AGE reduction. A reduction in serum CRP in patients with T2DM and VCAM-1 in participants without T2DM was also observed after intake of low AGE diets. A systematic review by Kellow *et al*.^14^ have also conducted meta-analyses for few markers of inflammation and oxidative stress and showed that AGE-restricted diet reduces TNFα and 8-isoprostanes in healthy adults, and VCAM-1 in patients with CKD. The observed reduction in markers of inflammation and oxidative stress together with an increased in adiponectin and sirtuin-1 and decreased leptin levels may be a major factor in improving insulin resistance in humans^39, 40^.

Regarding circulating AGEs, serum carboxymethyl-lysine has been shown to reduce after intake of low AGE diets in both individuals with and without T2DM^13, 14^. Related to this, our analyses have also showed that low AGE diets decreased serum carboxymethyl-lysine, methylglyoxal and receptor for AGE regardless of participants’ T2DM status and length of follow up. An increase in AGER1 after low AGE diets was observed only in patients with T2DM. AGEs induce effects within the body by causing structural changes of body proteins and by binding to cell surface receptors, such as receptor for AGE. Binding of receptors activates multiple signalling pathways, generates oxidative stress, induces vascular cell dysfunction, and enhances membrane protein expression and cytokine production^1^, which is in part manifested by increased expression of various pro-inflammatory molecules, tissue factor production, and interleukin expression^41^. The improvement of circulating AGEs and its transcription factors as showed in the current study has likely contributed to the suppression of inflammation and oxidative stress^17, 21^.

### Strength and limitations

In this paper, we have conducted meta-analyses on a broad range of cardiometabolic parameters including measures of insulin resistance, HbA1c, fasting and 2-h glucose and insulin from OGTT, cardiovascular risk factors and renal function in both individuals with and without T2DM. Both parallel and crossover randomised trials written in English language were included. Meta-analysis was performed for most of the listed outcomes. We were able to conduct meta-analyses of clinical cardiometabolic parameters that have not been reported before. We have included 560 participants (24 articles) in our systematic review compared to 289 participants (16 articles) in the previous review^14^. In addition, we have also conducted subgroup analyses to further explore the results of each parameter. Despite the above strengths, approximately half of the included studies had poor methodological quality, and we were unable to perform meta-analysis for hard clinical outcomes such as T2DM, CVD, stroke and death as there were no studies reporting on these parameters. The majority of the included studies had short duration of follow up and small number of participants. Meta-regression and publication bias was not conducted because of the small number of studies included in each meta-analysis. Future direction should include larger high quality randomised clinical trials with longer length of follow up and also assess hard clinical outcomes, such as the incidence and mortality of chronic disease. Differences in diets including baseline dietary AGE levels, methods and reliability of food preparation amongst studies must also be considered as a confounding factor.

In conclusion, our meta-analyses have shown that low AGE diets can reduce cardiometabolic risk factors such as insulin resistance, lipid profile, and markers of inflammation and oxidative stress. Importantly, low AGE diet is a low cost, safe, readily scalable and easy to implement strategy which can be used for both prevention and treatment of cardiometabolic risk factors and disease. Our findings could have important public health implications, such as for programs that promote sustainable low AGE consumption, influence guidelines for food preparation and processing. They could provide a guide for patients at risk for T2DM and CVD as well as add on to therapy for these diseases.

## Methods

### Data Sources and Searches

Electronic databases (MEDLINE, Embase, Scopus, Cochrane, CINAHL, and ProQuest) were searched up to May 2016 using a search strategy that combined terms for “diets”, “AGEs”, and “RCTs”. The full search strategy is provided in the Supplementary Table 4. Reference lists of all relevant studies and reviews, and the International Clinical Trials Registry Platform Search Portal (http://apps.who.int/trialsearch/) were searched.

### Study Selection

Only randomised controlled trials, both parallel and crossover trials of any sample size and duration conducted in humans were included. No specific exclusions were imposed to select the population of interest. The intervention consisted of at least one randomised group following a diet restricted in AGEs defined as: 1) Containing less than the amount of measured AGEs present in an unmodified diet high in AGEs, by any amount or 2) Containing less than the amount of measured AGEs present in a modified diet high in AGEs, by any amount or 3) Cooking or processing methods altered in such a way that is known to produce a reduced amount of AGEs. All of the studies had a comparison group consisting of people with diets high in AGEs defined as: 1) Unmodified diet containing higher amounts of measured AGEs compared to the low AGE diet by any amount or 2) Specifically modified diet containing higher amounts of measured AGEs compared to the low AGE diet by any amount or 3) Cooking or processing methods altered in a way that is known to produce higher amounts of AGEs by any amount.

The outcomes of interest are mentioned in Supplementary Table 5. In brief, primary outcomes included measures of glucose homeostasis, cardiovascular risk factors, and endpoint/hard clinical outcomes such as incidence of T2DM, CVD and CKD. Markers of inflammation, oxidative stress, endothelial dysfunction, and circulating AGEs as well as renal functions were considered as secondary outcomes.

No restrictions were imposed either on year of publication or publication status but the language was limited to English.

One clinical reviewer (VK), under the supervision of another author with extensive experience in conducting systematic reviews and meta-analysis (LM) screened the titles and abstracts of the records. Full articles were retrieved for further assessment if the study met the inclusion criteria. Where there was any doubt about inclusion, the study was reviewed and discussed with other reviewers to reach consensus.

### Data Extraction and Quality Assessment

Data were extracted by two independent reviewers (EB, VK) using a priori developed data extraction form. Information extracted included description of study (authors, country, year of publication, inclusion and exclusion criteria, primary and secondary outcomes, sample size), participants (number, age, gender, BMI), health status, comorbidities, intervention and control (type of dietary modification, dAGE content, length of follow up) and study results (endpoint or change in mean and standard deviations). In randomised crossover studies, we extracted the overall data based on the assumption that there was no carry over effect, that authors employed proper statistical tests to assess its effect, and that the reported results were comparable to parallel studies. When the data of interest were not available in the published paper, authors were contacted for further data.

Two independent reviewers (EB, VK) assessed the risk of bias of each included study using a descriptive component approach. Individual quality items including method of randomisation, allocation concealment, blinding to participants, investigators and outcome assessors, dropout rates, intention to treat analysis, selective outcome reporting, power of the study, washout period (for crossover studies) and method of statistical analysis were investigated. Any disagreements were resolved by discussion among reviewers. Based on this quality assessment, each study was rated as low, medium, high and insufficient information.

### Data Synthesis and Meta-Analysis

Statistical analyses were performed using Review Manager 5.3. Clinically and statistically homogeneous studies were pooled together and analysed using fixed-effects modelling (Mantel-Haenszel methods). Where data were clinically or statistically heterogeneous, data were combined for meta-analysis (random effects model) to provide pooled estimates on the efficacy of the interventions. Data are presented as mean difference (MD) with 95% confidence interval (CI). Statistical homogeneity was assessed using the I^2^ test with values over 50% indicating moderate to high heterogeneity^42^. Subgroup analysis was also performed comparing outcomes by health status (patients with T2DM/non-T2DM), dAGE content (high AGE/low AGE: <2/≥2 times), and length of follow up (<2/2-4/≥4weeks). Meta-regression was also planned for outcomes that were reported by at least ten studies.

### Data Availability

All data analysed during this study are included in this published article (and its Supplementary Information files). PROSPERO Registration Number: CRD42015023955.

## Electronic supplementary material

Supplementary Information File
