# Supplementary material for: Consumption of diets with low advanced glycation end products improves cardiometabolic parameters: meta-analysis of randomised controlled trials
Source: Sci Rep. 2017 May 23;7:2266. doi: 10.1038/s41598-017-02268-0 (PMC5442099; doi:10.1038/s41598-017-02268-0)
Supplement: Supplementary file 1 — Supplementary Information File [file 41598_2017_2268_MOESM1_ESM.pdf]

Running title: AGEs and cardiometabolic risk

Consumption of diets with low advanced glycation end products improves cardiometabolic parameters: meta-analysis of randomised controlled trials

Estifanos Baye[1*], Velislava Kiriakova[1*], Jaime Uribarri[2], Lisa J Moran[1], Barbora de Courten[1]

[1] Monash Centre for Health Research and Implementation, School of Public Health and Preventive Medicine, Monash University, 43-51 Kanooka Grove, Clayton, VIC 3168, Australia

[3] Department of Medicine, The Mount Sinai School of Medicine, One Gustave Levy Place, New York, NY 10029, USA

* Equal contribution

Supplemental Table 1: Brief characteristics of included studies

Study ID	Study design	Setting	Population	Sample size	Intervention	Control	dAGE content	Duration
Birlouez-Aragon 2010 [15]	Cross over	LaSalle Beauvais Polytechnical Institute - research centre setting	Healthy students aged >18 years and BMI of < 30 kg/m^2	64 randomised, 62 assessed	Steamed diet (low MRPs)	Standard diet (rich in MRPs)	HAGE = 5.4 + 2.3, LAGE = 2.2 + 0.9 mg CML/day	4 weeks
Cai 2004 [24]	Parallel	Mount Sinai School of Medicine General Clinical Research Centre	Diabetic patients with normal lipid profile & renal function	24 randomised: 11 HAGE & 13 LAGE	LAGE diet	HAGE diet	HAGE = 5 times higher than LAGE	6 weeks
de Courten 2016 [16]	Cross over	Baker IDI Heart and Diabetes Institute	Healthy but overweight individuals	28 randomised, 20 assessed	LAGE diet	HAGE diet	HAGE = 59, LAGE = 49 mg CML/day	2 weeks
Seiquer 2008 [33]<br>Delgado-Andrade 2012 [30]<br>Seiquer 2014 [32]	Cross over	San Cecilio University Hospital of Granada	Healthy male adolescents 11–14 years	20 randomised, 18 assessed	MRP-low diet: each of the 7-day menus was repeated in the 2nd week	MRP-high diet: each of the 7-day menus was repeated in the 2nd week	HAGE = 11.28 ± 0.27, LAGE = 5.36 ± 0.14 CML mg/day	2 weeks
Harcourt 2011 [18]	Cross over	Baker IDI Heart and Diabetes Institute	Overweight males aged 18 – 50 years and BMI 26 - 39kg/m2)	11 randomised, 11 assessed	LAGE diet	HAGE diet	HAGE = 14,090, LAGE = 3302 kU AGE/day	2 weeks

Luevano-Contreras 2013 [25]	Parallel	Department of medical science of the university of Guanajuato	Patients with type 2 diabetes	34 randomised, 26 assessed: 13 HAGE and 13 LAGE diet	LAGE diet	HAGE (standard) diet	HAGE = 9910 ± 4164, LAGE = 8956 ± 3587 kU CML/day	6 weeks
Macías-Cervantes 2015 [26]	Parallel	Department of Medical Sciences of The University of Guanajuato	Overweight men (BMI >25 kg/m^2) aged 30 to 55 y	75 randomised, 45 assessed: 15 in the diet plus exercise group, 14 in the exercise group, and 14 in the diet group	LAGE diet with exercise	Habitual diet with exercise	HAGE = 13284 ± 4983, LAGE = 13019 ± 4526 kU AGE/day	12 weeks
Mark 2014 [19]	Parallel	Department of nutrition, exercise and sports, university of Copenhagen	Overweight women aged 20–50 years	99 randomised, 74 assessed: 36 LAGE & 37 HAGE diet	LAGE diet either with 20 g fructose or 22 g glucose suppleme nts	HAGE diet either with 20 g fructose or 22 g glucose supplemen ts	HAGE = 24.6, LAGE = 10.7 mg CML/day	4 weeks
Poulsen 2014 [31]	Cross over	Department of nutrition, exercise and sports, university of Copenhagen	Healthy overweight individuals	20 randomised, 19 assessed	LAGE diet	HAGE diet	HAGE = 5, LAGE = 2.8 mg CML	05 hours
Semba 2014 [27]	Parallel	Beltsville Human Nutrition Research Center	Healthy adults aged 50–69 years	24 randomised, 24 assessed: 12 in each group	LAGE diet	HAGE diet (4 times higher in AGEs than the low-AGE diet)	HAGE = 4 times higher than LAGE	6 weeks

Striban 2007 [37]<br>Negrean 2007 [10]<br>Striban 2008a [36]<br>Striban 2008b [35]<br>Striban 2013 [34]	Cross over	Heart and Diabetes Center North Rhine-Westphalia	People with type 2 diabetes mellitus aged 41–71 years	20 randomised, 20 assessed	LAGE diet	HAGE diet	HAGE = 15100, LAGE = 2750 kU AGE/day	6 days
Uribarri 2003 [28]<br>Peppa 2004 [20]	Parallel	Mount Sinai Hospital Dialysis Service	Patients on peritoneal dialysis patients without diabetes	26 randomised, 18 assessed: 13 in each group	LAGE diet	HAGE diet	HAGE = 17000 ± 3700, LAGE = 5500 ± 900 kU CML/day	4 weeks
Uribarri 2011 [17]	Parallel	Mount Sinai School of Medicine	Type 2 diabetic patients and healthy subjects	18 randomised, 18 assessed: 12 LAGE & 6 HAGE	LAGE diet (AGE-restricted, <10 AGE Eq/day)	HAGE (standard, (>20 AGE Eq/day) diet	HAGE = > 20, LAGE = < 10 AGE Eq/day	4 months
Uribarri 2014 [21]	Parallel	Mount Sinai School of Medicine	Healthy participants over the age of 60	18 randomised, 18 assessed: 10 LAGE & 6 HAGE	LAGE diet	HAGE (regular) diet	HAGE = > 15, LAGE = < 10 AGE Eq/day	4 months
Vlassara 2002 [22]	Parallel + Cross over	Mount Sinai School of Medicine	Diabetic subjects with good glycaemic control and normal renal function	11 for cross over and 13 for parallel trials (6 in high and 7 in low AGE diet)	LAGE diet	HAGE diet	HAGE = 5 times higher than LAGE	Parallel 6 weeks, Cross over 2 weeks
Vlassara 2009 [29]	Parallel	Mount Sinai School of Medicine	Healthy subjects and patients with CKD	30 healthy subjects and 9 CKD-3 pts	LAGE diet	HAGE diet (dietary AGE	LAGE = 30 – 50% lower than HAGE	Healthy 4 months, patients with CKD

						intake >13 AGE Eq/d)		for 4 weeks
Vlassara 2016 [23]	Parallel	Mount Sinai School of Medicine	Obese subjects with metabolic syndrome aged 50 years or above	138 randomised, 100 assessed: 51 in LAGE & 49 HAGE	LAGE diets	Standard (usual) diet	HAGE = 20±11, LAGE = 7±6 AGE Eq/day	1 year

HAGE, high advanced glycation end products; LAGE, low advanced glycation end products

Supplemental Table 2: Risk of bias assessment for included studies

Study ID	Adequate randomisation	Concealed allocation	Blinding patients	Blinding care providers	Blinding outcome assessors	Dropout rate (%)	Intention to treat	Self-reporting bias	Sufficient power	Adequate wash-out period	Overall RoB
Birlouez-Aragon 2010 [15]	NR	NR	N	NR	NR	3.13 for each group	N	N	NR	10D – 4 W	Insufficient
Cai 2004 [24]	NR	NR	N	NR	NR	zero	Y	N	NR	N/A	Insufficient
de Courten 2016 [16]	Y	Y	Y	Y	Y	zero	N	N	NR	4W- 2WK	Low
Delgado-Andrade 2012 [30]<br>Seiquer 2008 [33]<br>Seiquer 2014 [32]	NR	NR	N	NR	NR	10% for both groups	N	N	NR	40D – 2W	Insufficient
Harcourt 2011 [18]	NR	NR	N	NR	NR	zero	Y	N	NR	4W – 2W	Insufficient
Luevano-Contreras 2013 [25]	Y	NR	N	Y	Y	23.5% for each group	N	N	NR	NA	Moderate
Macías-Cervantes 2015 [26]	Y	Y	N	Y	Y	2.7% total	N	N	Y	NA	Low
Mark 2014 [19]	Y	N	N	N	NR	25.3 total	N	N	Y	NA	Moderate
Poulsen 2014 [31]	Y	Y	N	N	Y	5% total	N	N	Y	5H – 2W	Low
Semba 2014 [27]	Y	Y	Y	Y	Y	zero	Y	N	Y	N/A	Low
Striban 2007 [37]<br>Negrean 2007 [10]<br>Striban 2008a [36]<br>Striban 2008b [35]<br>Striban 2013 [34]	NR	NR	N	Y	NR	zero	Y	N	Y	7D – 6D	Moderate
Uribarri 2003 [28]<br>Peppa 2004 [20]	NR	NR	N	NR	NR	30.8% for each group	N	N	NR	NA	Insufficient
Uribarri 2011 [17]	NR	NR	N	NR	NR	zero	Y	N	NR	N/A	Insufficient

Uribarri 2014 [21]	NR	NR	N	NR	NR	zero	Y	N	NR	N/A	Insufficient
Vlassara 2002 [22]	NR	NR	N	NR	NR	zero	Y	N	NR	N/A – parallel; 1/2W – 2W - crossover	Insufficient
Vlassara 2009 [29]	NR	NR	N	NR	NR	6.6% total	Y	N	NR	N/A	Insufficient
Vlassara 2016 [23]	NR	NR	N	N	Y	19.6% HAGE, 33.7% LAGE	N	N	Y	N/A	Moderate

Supplemental Table 3: Subgroup analyses for cardiometabolic parameters

Parameters		Participants' T2DM status		dAGE content (high/low AGE)		Length of follow up (weeks)	
		No	Yes	≥ 2 times	< 2 times	2 - 4	≥ 4
Fasting glucose (mg/dl)	MD (95% CI)	-0.4 (-2.3, 1.5)	-12.2 (-39.3, 14.8)	-2.3 (-7.9, 3.1)	0.4 (-2.2, 3.1)	0.2 (-4.1, 4.5)	-0.3 (-3, 2.5)
	P-value	0.7	0.4	0.4	0.7	0.9	0.8
HbA1c (%)	MD (95% CI)	0.05 (-0.9, 1.0)	-0.00 (-0.09, 0.08)	NA		NA	
	P-value	0.9	0.9				
Fasting insulin (µU/ml)	MD (95% CI)	-1.8 (-4.4, 0.9)	-7 (-11.5, -2.5)	-2.5 (-5.7, 0.7)	-2 (-4.8, 0.8)	-2 (-4.8, 0.8)	-2.5 (-5.7, 0.7)
	P-value	0.2	0.002	0.1	0.1	0.1	0.1
HOMA-IR	MD (95% CI)	-0.5 (-1, -0.03)	-4.9 (-5.5, -4.3)	-1.8 (-3.6, 0.02)	-0.3 (-0.8, 0.2)	-0.6 (-1.1, -0.07)	-1.4 (-2.6, -0.2)
	P-value	0.04	0.001	0.05	0.2	0.03	0.02
Weight (kg)	MD (95% CI)	-0.23 (-3.9, 3.5)	-7.6 (-24.9, 9.6)	-2.5 (-6.7, 1.6)	2.9 (-3.9, 9.7)	-0.5 (-6.6, 5.6)	-0.8 (-6.1, 4.5)
	P-value	0.9	0.4	0.2	0.4	0.8	0.7
BMI (kg/m2)	MD (95% CI)	-0.5 (-1.9, 0.9)	0.00 (-14.7, 14.7)	NA		0.09 (-3.2, 3.4)	-0.6 (-2.2, 1.1)
	P-value	0.5	1			0.9	0.5
Systolic BP (mm Hg)	MD (95% CI)	1.62 (-2.4, 5.6)	1.2 (-10.5, 12.9)	1.3 (-2.6, 5.3)	3.5 (-6.3, 13.3)	7 (-4.6, 18.6)	0.9 (-2.8, 4.8)
	P-value	0.4	0.8	0.5	0.4	0.2	0.6
Diastolic BP (mm Hg)	MD (95% CI)	2.2 (-0.6, 5)	-0.3 (-6.5, 5.9)	2 (-0.7, 4.8)	0.4 (-6.4, 7.2)	-1 (-8.7, 6.7)	2.1 (-0.6, 4.8)
	P-value	0.1	0.9	0.1	0.9	0.8	0.1
Total cholesterol (mg/dl)	MD (95% CI)	-8.5 (-9.5, -7.4)	-5.3 (-27, 17)	NA		-2.2 (-22, 18)	-8.5 (-9.6, -7.4)
	P-value	0.001	0.6			0.8	0.001
LDL (mg/dl)	MD (95% CI)	-3.3 (-8.3, 1.8)	-4.3 (-19, 11)	-2.4 (-3.5, -1.3)	-0.2 (-15, 15)	-3.8 (-20, 13)	-2.4 (-3.4, -1.2)
	P-value	0.2	0.6	0.001	0.9	0.6	0.001
HDL (mg/dl)	MD (95% CI)	-3.8 (-11.7, 4.1)	-1.1 (-7.2, 5.1)	-1.9 (-7.6, 3.8)	-1.4 (-12.8, 9.9)	-3.9 (-13.1, 5.3)	-1.3 (-6.9, 4.3)
	P-value	0.8	0.1	0.5	0.8	0.9	0.3
Triglycerides (mg/dl)	MD (95% CI)	-12.7 (-31.6, 6.2)	0.39 (-17, 16.3)	-7.7 (-22.6, 7.2)	-9.9 (-42.8, 22.9)	8.8 (-15.6, 33.2)	-10.5 (-24.7, 3.6)
	P-value	0.2	0.9	0.3	0.6	0.5	0.1
CRP (mg/dl)	MD (95% CI)	-0.01 (-0.12, 0.11)	-1.22 (-2.27, -0.18)	-0.5 (-1.52, 0.51)	0.1 (-0.21, 0.41)	-0.02 (-0.15, 0.1)	-0.48 (-1.63, 0.66)

	P-value	0.9	0.02	0.33	0.5	0.72	0.41
TNFα (ng/mg)	MD (95% CI)	-4.6 (-6.5, -2.8)	-6.2 (-15, -2.4)	-4.9 (-7.6, -2.1)	-3.9 (-6.7, -1.1)	NA	
	P-value	0.001	0.001	0.001	0.006		
Adiponectin (μg/ml)	MD (95% CI)	8.2 (6.2, 10.3)	5.8 (3.8, 7.8)	7.1 (5.6, 8.5)	2.1 (-10.2, 14.4)		
	P-value	0.001	0.001	0.001	0.7	NA	
VCAM-1 (ng/ml)	MD (95% CI)	-250 (-343, -157)	-215 (-512, 82)	-193 (-302, -83)	-286 (-423, -149)	-410 (-736, -83)	-200 (-295, -105)
	P-value	0.001	0.2	0.001	0.001	0.01	0.001
8-Isoprostane (pg/ml)	MD (95% CI)	-103 (-167, -38)	-172 (-326, -17)	-128 (-208, -48)	-70 (-123, -17)	NA	
	P-value	0.002	0.03	0.002	0.009		
Serum CML (U/ml)	MD (95% CI)	-3.8 (-6, -1.9)	-10.6 (-14, -7.1)	-6.9 (-10, -3.7)	-2.5 (-4, -0.8)	NA	
	P-value	0.001	0.001	0.001	0.002		
Serum MG (nmol/ml)	MD (95% CI)	-0.3 (-0.4, -0.2)	-1.6 (-2.1, -1)	-0.6 (-1, -0.07)	-0.3 (-0.6, -0.1)	NA	
	P-value	0.01	0.03	0.02	0.04		
sRAGE (mRNA)	MD (95% CI)	-173.4 (-305, -42.8)	-420 (-674, -166)	-173.4 (-305, -42.8)	-420 (-674, -166)	NA	
	P-value	0.009	0.001	0.009	0.001		
AGER1 (mRNA)	MD (95% CI)	-20.2 (-112, 72)	96 (46, 146)	42 (-17, 100)	-96.5 (-208, 15)	NA	
	P-value	0.7	0.002	0.2	0.09		
SERT1 (mRNA)	MD (95% CI)	180 (94, 265)	204 (76, 332)	177 (91, 263)	210 (90, 330)	NA	
	P-value	0.001	0.002	0.001	0.001		

Random effects model was used.

AGER1, advanced glycation endproduct receptor 1; BMI, body mass index; BP, blood pressure; CI, confidence interval; CML, carboxymethyl lysine; CRP, c-reactive protein; eGFR, estimated glomerular filtration rate; HDL, high density lipoprotein; HbA1c, haemoglobin A1c; HOMA-IR, homeostatic model of insulin resistance; Insulin AUC, insulin area under the curve; MD, mean difference; MG, methylglyoxal; P, p-value; SERT-1, sertoli cell protein 1; sRAGE, soluble form of receptor for advanced glycation endproducts; TNF, tumour necrosis factor; VCAM-1, vascular cell adhesion protein 1.

Supplemental Table 4 - Database Search strategy
All of the database searches were finalised and completed by the 10/05/2016

Ovid Medline Search strategy
1. exp Diet/
2. exp Food/
3. exp Eating/
4. Nutritive Value/
5. nutrition therapy/ or diet therapy/
6. exp Meals/
7. Energy Intake/
8. diet*.mp.
9. food*.mp.
10. (food adj2 restrict*).mp.
11. food regimen*.mp.
12. nutrition.mp.
13. nutrition* therap*.mp.
14. diet* regime*.mp.
15. diet* program*.mp.
16. diet* therap*.mp.
17. meal*.mp.
18. (meal adj2 restrict*).mp.
19. energy intake.mp.
20. calorie intake.mp.
21. (food adj2 modif*).mp.
22. (meal adj2 modif*).mp.
23. (nutrient* adj2 intake).mp.
24. 1 or 2 or 3 or 4 or 5 or 6 or 7 or 8 or 9 or 10 or 11 or 12 or 13 or 14 or 15 or 16 or 17 or 18 or 19 or 20 or 21 or 22 or 23
25. Glycosylation End Products, Advanced/
26. glycosylation/ or maillard reaction/
27. advanced glyc*.mp.
28. maillard.mp.
29. (browning adj2 reaction).mp.
30. high oxidant compounds.mp.
31. (nepsilon adj2 carboxymethyllysine).mp.
32. (methyl adj2 glyoxal).mp.
33. advanced lipox*.mp.
34. glycat* stress.mp.
35. soluble receptor of advanced glycation end products.mp.
36. sRAGE.mp.
37. endogenous secretory receptor for advanced glycation end products.mp.
38. esRAGE.mp.
39. glycation.mp.
40. thermal.mp.
41. 25 or 26 or 27 or 28 or 29 or 30 or 31 or 32 or 33 or 34 or 35 or 36 or 37 or 38 or 39 or 40
42. 24 and 41
43. randomised control trial*.mp.
44. randomized control trial*.mp.
45. RCT*.mp.

46. randomized controlled trial.mp.
47. randomised controlled trial.mp.
48. controlled clinical trial*.mp.
49. random allocation.mp.
50. double blind method*.mp.
51. single blind method*.mp.
52. randomised.mp.
53. randomized.mp.
54. randomisation.mp.
55. randomization.mp.
56. placebo.mp.
57. drug therapy.fs.
58. randomly.ab.
59. trial.ab.
60. 43 or 44 or 45 or 46 or 47 or 48 or 49 or 50 or 51 or 52 or 53 or 54 or 55 or 56 or 57 or 58 or 59
61. 42 and 60
62. animals.mp. not (humans and animals).sh.
63. 61 not 62

exp= exploded MESH
mp=title, abstract, original title, name of substance word, subject heading word, keyword heading word, protocol supplementary concept word, rare disease supplementary concept word, unique identifier
*= Substitute one, or no characters

ProQuest
mesh.Exact("Meals" OR "Food" OR "Diet") OR ALL(diet*) OR ALL(food*) OR ALL(food NEAR/2 restriction) OR ALL(food regimen*) OR ALL(nutrition) OR ALL(nutrition* therap*) OR ALL(diet* regime*) OR ALL(diet* program*) OR ALL(diet* therap*) OR ALL(meal*) OR ALL(meal NEAR/2 restrict*) OR ALL(energy intake) OR ALL(nutrients NEAR/2 intake) OR ALL(calorie intake) OR ALL(food NEAR/2 modif*) OR ALL(meal NEAR/2 modif*)
AND
mesh.Exact("Glycosylation End Products, Advanced" OR "Maillard Reaction") OR ALL(advanced glyc*) OR ALL(maillard) OR ALL(browning NEAR/2 reaction) OR ALL(high oxidant compounds) OR ALL(nepsilon NEAR/2 carboxymethyllysine) OR ALL(methyl NEAR/2 glyoxal) OR ALL(advanced lipox*) OR ALL(glycat* stress) OR ALL(soluble receptor of advanced glycation end products) OR ALL(sRAGE) OR ALL(endogenous secretory receptor for advanced glycation end products) OR ALL(esRAGE) OR ALL(glycation) or ALL(thermal)
AND
ALL(randomised control trial*) OR ALL(randomized control trial*) OR ALL(RCT*) OR ALL(randomised controlled trial*) OR ALL(randomized controlled trial*) OR ALL(controlled clinical trail*) OR ALL(random allocation) OR ALL(double blind method*) OR ALL(single blind method*) OR ALL(randomised) OR ALL(randomized) OR ALL(randomisation) OR ALL(randomization) OR ALL(placebo) OR AB(drug therapy) OR AB(randomly) OR AB(trial)

Embase
Full text search limit RCT and human
[mp=title, abstract, heading word, drug trade name, original title, device manufacturer, drug manufacturer, device trade name, keyword]

1. exp nutrition/
2. diet*.mp.
3. food*.mp.
4. (food adj2 restrict*).mp.
5. food regimen*.mp.
6. nutrition.mp.
7. nutrition* therap*.mp.
8. diet* regime*.mp.
9. diet* program*.mp.
10. diet* therap*.mp.
11. meal*.mp.
12. (meal adj2 restrict*).mp.
13. energy intake.mp.
14. calorie intake.mp.
15. (food adj2 modif*).mp.
16. (meal adj2 modif*).mp.
17. (nutrient* adj2 intake).mp.
18. 1 or 2 or 3 or 4 or 5 or 6 or 7 or 8 or 9 or 10 or 11 or 12 or 13 or 14 or 15 or 16 or 17
19. exp glycosylation/
20. advanced glycation end product/ or advanced glycation end product receptor/
21. advanced glyc*.mp.
22. maillard.mp.
23. (browning adj2 reaction).mp.
24. (nepsilon adj2 carboxymethyllysine).mp.
25. (methyl adj2 glyoxal).mp.
26. advanced lipox*.mp.
27. glycat* stress.mp.
28. soluble receptor of advanced glycation end products.mp.
29. sRAGE.mp.
30. endogenous secretory receptor for advanced glycation end products.mp.
31. esRAGE.mp.
32. glycation.mp.
33. thermal.mp.
34. 19 or 20 or 21 or 22 or 23 or 24 or 25 or 26 or 27 or 28 or 29 or 30 or 31 or 32 or 33
35. 18 and 34
36. limit 35 to (clinical trial or randomized controlled trial or controlled clinical trial or multicenter study)
37. clinical trial*.mp.
38. random*.tw.
39. placebo.tw.
40. (doubl* adj blind*).mp.
41. (singl* adj blind*).mp.
42. ((treble or triple) adj blind*).mp.
43. (crossover* or cross-over*).mp.

44. 36 or 37 or 38 or 39 or 40 or 41 or 42 or 43
45. 18 and 34 and 44
46. exp animal/ not human.sh.
47. 45 not 46

Cochrane central
AGEs systematic review cochrane central final 170915
full text search limit RCT and human
[mp=title, original title, abstract, mesh headings, heading words, keyword]

1. exp Diet/
2. exp Food/
3. exp Eating/
4. Nutritive Value/
5. nutrition therapy/ or diet therapy/
6. exp Meals/
7. Energy Intake/
8. diet*.mp.
9. food*.mp.
10. (food adj2 restrict*).mp.
11. food regimen*.mp.
12. nutrition.mp.
13. nutrition* therap*.mp.
14. diet* regime*.mp.
15. diet* program*.mp.
16. diet* therap*.mp.
17. meal*.mp.
18. (meal adj2 restrict*).mp.
19. energy intake.mp.
20. calorie intake.mp.
21. (food adj2 modif*).mp.
22. (meal adj2 modif*).mp.
23. (nutrient* adj2 intake).mp.
24. 1 or 2 or 3 or 4 or 5 or 6 or 7 or 8 or 9 or 10 or 11 or 12 or 13 or 14 or 15 or 16 or 17 or 18 or 19 or 20 or 21 or 22 or 23
25. Glycosylation End Products, Advanced/
26. glycosylation/ or maillard reaction/
27. advanced glyc*.mp.
28. maillard.mp.
29. (nepsilon adj2 carboxymethyllysine).mp.
30. soluble receptor of advanced glycation end products.mp.
31. sRAGE.mp.
32. endogenous secretory receptor for advanced glycation end products.mp.
33. esRAGE.mp.
34. glycation.mp.
35. thermal.mp.
36. 25 or 26 or 27 or 28 or 29 or 30 or 31 or 32 or 33 or 34 or 35
37. 24 and 36
38. randomised control trial*.mp.

39. randomized control trial*.mp.
40. RCT*.mp.
41. randomized controlled trial.mp.
42. randomised controlled trial.mp.
43. controlled clinical trial*.mp.
44. random allocation.mp.
45. double blind method*.mp.
46. single blind method*.mp.
47. randomised.mp.
48. randomized.mp.
49. randomisation.mp.
50. randomization.mp.
51. placebo.mp.
52. drug therapy.fs.
53. randomly.ab.
54. trial.ab.
55. 38 or 39 or 40 or 41 or 42 or 43 or 44 or 45 or 46 or 47 or 48 or 49 or 50 or 51 or 52 or 53 or 54
56. 37 and 55
57. (animals not (humans and animals)).mp. [mp=title, original title, abstract, mesh headings, heading words, keyword]
58. 56 not 57

SCOPUS
((TITLE-ABS-KEY (**diet***) OR TITLE-ABS-KEY (**food***) OR TITLE-ABS-KEY (**food** W/2 **restrict***) OR TITLE-ABS-KEY (**food regimen***) OR TITLE-ABS-KEY (**nutrition**) OR TITLE-ABS-KEY (**nutrition* therap***) OR TITLE-ABS-KEY (**diet* regime***) OR TITLE-ABS-KEY (**diet* program***) OR TITLE-ABS-KEY (**diet* therap***) OR TITLE-ABS-KEY (**meal***) OR TITLE-ABS-KEY (**meal** W/2 **restrict***) OR TITLE-ABS-KEY (**energy intake**) OR TITLE-ABS-KEY (**calorie intake**) OR TITLE-ABS-KEY (**food** W/2 **modif***) OR TITLE-ABS-KEY (**meal** W/2 **modif***) OR TITLE-ABS-KEY (**nutrient*** W/2 **intake**))) AND ((TITLE-ABS-KEY (**advanced glyc***) OR TITLE-ABS-KEY (**maillard**) OR TITLE-ABS-KEY (**browning** W/2 **reaction**) OR TITLE-ABS-KEY (**high oxidant compounds**) OR TITLE-ABS-KEY (**nepsilon** W/2 **carboxymethyllysine**) OR TITLE-ABS-KEY (**methyl** W/2 **glyoxal**) OR TITLE-ABS-KEY (**advanced lipox***) OR TITLE-ABS-KEY (**glycat* stress**) OR TITLE-ABS-KEY (**soluble receptor of advanced glycation end products**) OR TITLE-ABS-KEY (**srage**) OR TITLE-ABS-KEY (**endogenous secretory receptor for advanced glycation end products**) OR TITLE-ABS-KEY (**esrage**) OR TITLE-ABS-KEY (**glycation**) OR TITLE-ABS-KEY (**thermal**))) AND ((TITLE-ABS-KEY (**randomised control trial***) OR TITLE-ABS-KEY (**randomized control trial***) OR TITLE-ABS-KEY (**rct***) OR TITLE-ABS-KEY (**randomised controlled trial***) OR TITLE-ABS-KEY (**randomized controlled trial***) OR TITLE-ABS-KEY (**controlled clinical trial***) OR TITLE-ABS-KEY (**random allocation**) OR TITLE-ABS-

KEY (**double blind method***) OR TITLE-ABS-KEY (**single blind method***) OR TITLE-ABS-KEY (**randomised**) OR TITLE-ABS-KEY (**randomized**) OR TITLE-ABS-KEY (**randomisation**) OR TITLE-ABS-KEY (**randomization**) OR TITLE-ABS-KEY (**placebo**) OR ABS (**drug therapy**) OR ABS (**randomly**) OR ABS (**trial**)))

CINAHL
TX = all text

1. (MH "Food+")
2. (MH "Diet+")
3. (MH "Eating")
4. (MH "Nutritive Value")
5. (MH "Nutrition Therapy (Iowa NIC)") OR (MH "Diet Therapy+")
6. TX diet*
7. TX food*
8. TX food N2 restrict*
9. TX food regimen*
10. TX nutrition
11. TX nutrition* therap*
12. TX diet* regime*
13. TX diet* program*
14. TX diet* therap*
15. TX meal*
16. TX meal N2 restrict*
17. TX energy intake
18. TX calorie intake
19. TX food N2 modif*
20. TX meal N2 modif*
21. TX nutrient* N2 intake
22. S1 OR S2 OR S3 OR S4 OR S5 OR S6 OR S7 OR S8 OR S9 OR S10 OR S11 OR S12 OR S13 OR S14 OR S15 OR S16 OR S17 OR S18 OR S19 OR S20 OR S21
23. (MH "Glycosylation")
24. TX advanced glyc*
25. TX maillard
26. TX browning N2 reaction
27. TX methyl N2 glyoxal
28. TX advanced lipox*
29. TX glycat* stress
30. TX soluble receptor of advanced glycation end products
31. TX sRAGE
32. TX endogenous secretory receptor for advanced glycation end products
33. TX esRAGE
34. TX glycation

35. TX thermal
36. S23 OR S24 OR S25 OR S26 OR S27 OR S28 OR S29 OR S30 OR S31 OR S32 OR S33 OR S34 OR S35
37. S22 AND S36
38. TX randomised control trial*
39. TX randomized control trial*
40. TX RCT*
41. TX randomized controlled trial
42. TX randomised controlled trial
43. TX controlled clinical trial*
44. TX random allocation
45. TX double blind method*
46. TX single blind method*
47. TX randomized
48. TX randomized
49. TX randomization
50. TX randomization
51. TX placebo
52. AB randomly
53. AB trial
54. S38 OR S39 OR S40 OR S41 OR S42 OR S43 OR S44 OR S45 OR S46 OR S47 OR S48 OR S49 OR S50 OR S51 OR S52 OR S53
55. S37 AND S54

Supplemental Table 5: List of outcomes of interest

Primary outcomes	Secondary outcomes
**1. Measures of glucose homeostasis**:<br>- fasting glucose, 2-hour glucose and insulin, fasting insulin, fasting C-peptide, HbA1c, HOMA-IR, HOMA-B, QUICKI & Matsuda Index, and glucose AUC, insulin AUC and C-peptide AUC after OGTT<br>2. **Cardiovascular risk factors:**<br>- weight, body mass index, waist circumference, blood pressure, pulse wave velocity, pulse wave analysis<br>- lipid profiles such as total cholesterol, HDL, LDL, triglycerides, free fatty acids<br>3. **Endpoint/hard clinical outcomes**<br>- T2DM, CVD, myocardial infarction, stroke & death	1. **Markers of inflammation**:<br>- C - reactive protein, tumour necrosis factor alpha, NF-kB activity and protein content, adiponectin, interleukin-6<br>2. **Oxidative stress**:<br>- 8-isoprotanes, urine isoprostane, ubiquinol, malondialdehyde, advanced oxidation products,<br>3. **Endothelial dysfunction**:<br>- vascular cell adhesion protein-, intercellular adhesion molecule-1, monocyte chemoattractant protein-1, flow mediated dilation<br>4. **Circulating AGEs**:<br>- total, serum, urine or faecal carboxyl methyl-lysine, methylglyoxal, methyl ethyl-lysine, receptor for advanced glycation end products, advanced glycation end products receptor -1<br>5. **Renal function**:<br>- estimated glomerular filtration rate, albumin-creatinine ratio, creatinine clearance, proteinuria and urine albumin
